# Relative Orientation and Position Detections Based on an RGB-D Sensor and Dynamic Cooperation Strategies for Jumping Sensor Nodes Recycling

**DOI:** 10.3390/s150923618

**Published:** 2015-09-17

**Authors:** Jun Zhang, Xi Yang, Guang-Ming Song, Tian-Yuan Chen, Yong Zhang

**Affiliations:** School of Instrument Science and Engineering, Southeast University, Nanjing 210096, China; E-Mails: yangxigreat@163.com (X.Y.); mikesong@seu.edu.cn (G.-M.S.); otischan@163.com (T.-Y.C.); 220142640@seu.edu.cn (Y.Z.)

**Keywords:** wireless sensor network, jumping robot, relative localization, relative orientation detection, Kinect, motion capture, dynamic cooperation, sensor node recycling, multi-robot docking

## Abstract

This paper presents relative orientation and position detection methods for jumping sensor nodes (JSNs) recycling. The methods are based on motion captures of the JSNs by an RGB-D sensor mounted on a carrier robot and the dynamic cooperation between the carrier and the JSNs. A disc-like label with two different colored sides is mounted on the top of the JSNs. The RGB-D sensor can detect the motion of the label to calculate the orientations and positions of the JSNs and the carrier relative to each other. After the orientations and positions have been detected, the JSNs jump into a cabin mounted on the carrier in dynamic cooperation with the carrier for recycling. The performances of the proposed methods are tested with a prototype system. The results show that the carrier can detect a JSN from up to 2 m away and sense its relative orientation and position successfully. The errors of the JSN’s orientation and position detections relative to the carrier could be reduced to the values smaller than 1° and 1 cm, respectively, by using the dynamic cooperation strategies. The proposed methods in this paper could also be used for other kinds of mobile sensor nodes and multi-robot systems.

## 1. Introduction

Wireless sensor networks (WSNs) can provide quick construction and easy utilization for measurement systems. WSNs also reduce the operation and maintenance costs of the systems. Wireless sensor nodes have been integrated into various application systems such as home automation [1], bridge health monitoring [2], forest fire monitoring [3], marine monitoring [4], volcano monitoring [5], agriculture [6], military [7], and space exploration [8], which cannot be built easily and conveniently in wired or the application environments are dangerous and inaccessible. Most of the sensor nodes in the systems are static sensor nodes without motion capability. However, if some sensor nodes in the network are not deployed well or their energy is exhausted, the whole WSN could lose functionality or even fail. 

In order to optimize the performance of a WSN, mobile robots are added into the WSN as mobile sink or sensor nodes [9], which form the mobile wireless sensor network (MWSN). The MWSN has some advantages compared to the WSN. Mobile sensor nodes (MSNs) can adjust their positions locally to dynamically optimize the network topology. This could contribute to improved coverage and overall network lifetime, reduced power consumption, superior channel capacity and better target tracking [9,10,11]. The MSNs are also able to move to the locations of damaged or energy exhausted sensor nodes to repair, recharge, or replace them, which could repair the interrupted network caused by the failed sensor nodes [12,13]. 

The mobile robots in a MWSN are usually small sized [14,15,16,17]. They are intended to be deployed and applied in outdoor environments. Small obstacles in these environments will limit the mobility of small-sized mobile robots which is the so called “scale effects” found in locomotion of animals and insects [18]. Hence, the traditional small wheeled robotic sensor nodes may not be used in outdoor uneven terrain such as areas with dense grass. Tall obstacles or deep ditches will also restrict the usage of small tracked and legged robotic sensor nodes. Miniature jumping robots inspired by creatures such as locusts [19], froghoppers [20], and fleas [21] can be adopted as MSNs, which could overcome obstacles several times taller or wider than their bodies. Sensor nodes with this locomotion capability can jump over obstacles or jump up onto the top of obstacles to improve signal quality and network connection of the MWSN [12]. The jumping sensor nodes (JSNs) are even able to improve network coverage when they perform airborne communications with each other [22]. 

Because the energy of the MSNs is usually supplied by batteries, they cannot traverse a very long distance for self-deployment. The sensor nodes in WSN and MWSN are usually transported and deployed by humans or by airplanes [23]. However, human deployment is not feasible for environments that humans cannot access. The MSNs could be carried by large wheeled or tracked carrier robots [24] for long-range transportation and deployment. In addition, the energy exhausted or damaged sensor nodes may result in waste and environment pollution if they are discarded [25]. The carrier robots can recycle the sensor nodes for recharging, damage repair, and redeploying. Being able to find the MSNs during recycling is very important for the carrier. Furthermore, the successful docking between the MSNs and the carrier is also crucial for MSNs recycling. 

The difficulties during recycling include relative orientation and position detections between the MSNs and the carrier, and the docking method design. The compass can be used for the orientation detection [24]. However, this kind of magnetic sensor is easily influenced by the motors and other electronic components of the MSNs [26]. The magnetic field may also be sheltered or obstructed in some environments. Small bodies such as asteroids have only weak magnetic fields [27]. The relative localization can be divided into long-distance localization and short-distance localization. For the long-distance localization, the GPS [28,29] and WSN [30,31,32] based localization methods could be utilized. However, these methods do not have high precision in short distance localization during MSNs recycling. Short-distance localization methods such as infrared [33], ultrasonic [34], RFID [35], and visual [36] based methods could be used to deal with this problem. 

The docking method design for MSNs recycling could adopt two main approaches. The first one is that the MSNs are grasped by the grasper of a manipulator mounted on the carrier [37]. The carrier needs a manipulator, which may be expensive. The other one is that the MSNs move into the recycling cabin of the carrier [38,39]. This method is simple and low cost, but needs dynamic cooperation between the MSNs and the carrier. Sensing and control between the two kinds of robots in some conditions are very tough things especially for JSNs [26]. The authors in [40,41,42] adopted visual detection methods based on static colored labels for wheeled robots docking. However, the static label is not suitable for the JSNs because they do not have the fine position adjustment capability like the wheeled robots. 

In this paper, we present JSNs recycling by a wheeled carrier robot, including short-distance orientation and position detection methods. The detection methods are based on an RGB-D sensor and dynamic cooperation strategies. The dynamic cooperation between the JSN and the carrier can improve orientation and position detection precisions and reduce the difficulties during docking. The rest of this paper is organized as follows. Section 2 introduces the components of the recycling system and its working procedure. Relative orientation and position detections based on the RGB-D sensor and dynamic cooperation strategies are investigated in Section 3. Prototype design and fabrication are described in Section 4. Experimental validations are conducted in Section 5. Conclusions and Future Work are given in Section 6. 

## 2. System Overview

### 2.1. Components of the Recycling System

**Figure 1:**
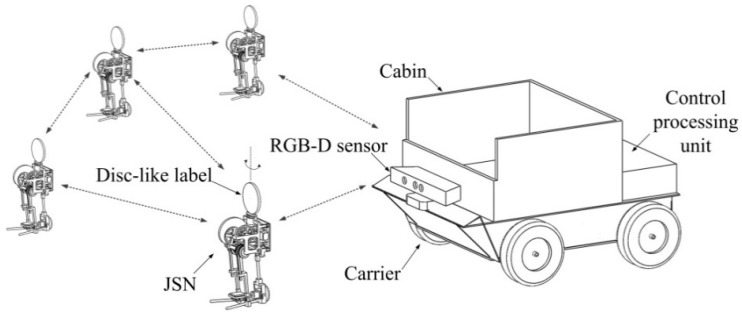
Components of the jumping sensor nodes recycling system.

The components of the proposed JSNs recycling system include a wheeled carrier and several miniature JSNs, as illustrated in Figure 1. The carrier and the JSNs form a mesh network in which the carrier is the coordinator and the JSNs are routers or end devices. The modeling, simulation, and system design of the JSN are presented in our previous work [43,44]. The JSN can jump about 1 m high and 0.65 m far. The JSN also has continuous locomotion capability. In this paper, a disc-like label is added on the top of the JSN and is driven by a stepper motor to rotate around the vertical axis. The two sides of the surface of the label have different colors, which are easy to visually detect and distinguish. An RGB-D sensor Kinect from Microsoft (Redmond, WA, USA) is mounted at the head of the carrier with the same height as the label on the JSN. The Kinect can detect the orientation of the label relative to the carrier and the distance between the JSN and the carrier. The control processing unit processes the image frames and depth information from the Kinect to calculate the relative orientation and position. The cabin on the top of the carrier is used for recycling the JSN. 

### 2.2. Assumptions and Working Procedure

We assume that the carrier can detect the position and battery information of the JSNs periodically and save them in its database during long-distance localization. The carrier is able to move to a JSN for recycling it when its power is lower than a threshold, which is the most common problem that may happen to JSNs. The carrier can also navigate to a JSN using the latest position information of the JSN to replace it if it is damaged suddenly. Because we focus the short-distance relative orientation and position detections in this paper, we assume that the initial distance between the JSN and the carrier is within 2 m after the carrier moves to the JSN using long-distance localization and navigation methods. 

**Figure 2:**
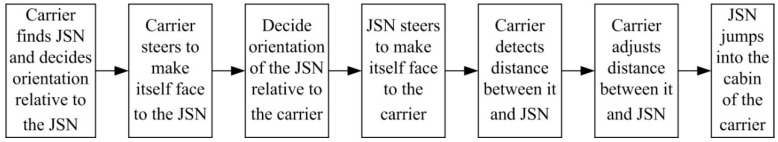
Working procedure of the jumping sensor node (JSN) recycling.

The working procedure of a JSN recycling is shown in Figure 2. The JSN recycling includes seven steps. Firstly, the carrier tries to find the JSN around it and decides the orientation of it relative to the JSN. Secondly, the carrier steers until it faces to the JSN, which means the heading of the carrier relative to the JSN is zero. Thirdly, the image frames from the Kinect will be processed by the carrier to calculate the orientation of the JSN relative to the carrier. Fourthly, the JSN steers to make itself face to the carrier. Fifthly, the carrier detects the distance between it and the JSN using depth sensor of the Kinect. Sixthly, the carrier adjusts the distance between it and the JSN to make sure that the JSN can jump into its cabin. Finally, the JSN jumps into the cabin. 

## 3. Relative Orientation and Position Detection Methods

**Figure 3:**
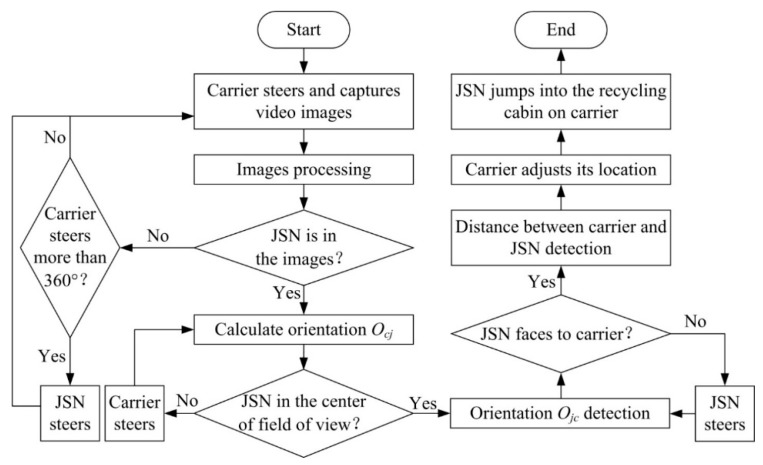
Flow chart of the relative orientations and position detections for JSN recycling.

The flow chart of the relative orientation and position detections is shown in Figure 3. The carrier steers and captures video images periodically using the Kinect. The images are processed using OpenCV (Open Source Computer Vision) to judge if the JSN is in the images. If the JSN is not in the images and its steering angle is smaller than 360°, the carrier will steer continuously. If the carrier steers more than 360° and still does not find the JSN, the JSN will steer a proper angle to help the carrier to find it. After finding that the JSN is in the field of view of the Kinect, the carrier calculates its orientation (*O_cj_*) relative to the JSN and judges if the JSN is in the center of the field of view. The carrier steers and calculates *O_cj_* until the JSN is in the center of the field of view. Then the carrier stops steering and detects the orientation *O_jc_* of the JSN relative to the carrier. The JSN steers if needed until it faces to the carrier. Next, the depth sensor is used to detect the distance between the carrier and the JSN. The carrier adjusts its location in order to make the cabin enter one jump range of the JSN. Finally, the JSN jumps into the cabin to finish the JSN recycling process. 

### 3.1. Orientation of Carrier Relative to JSN

**Figure 4:**
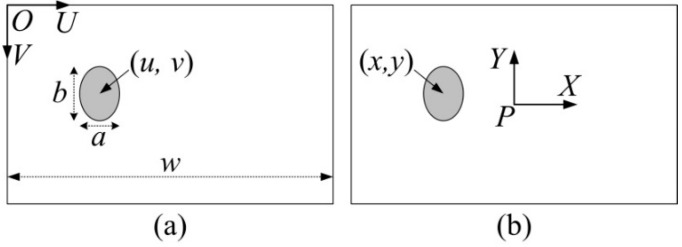
Diagram showing the disc-like label in the coordinate frames: (**a**) the pixel coordinate frame *U-O-V*; (**b**) the image plane coordinate frame *X-P-Y*.

The color camera of the Kinect is used to record objects around the carrier. The pixel coordinate frame *U-O-V* (a) and image plane coordinate frame *X-P-Y* (b) are shown in Figure 4. The carrier tries to find the label on the JSN. In order to decide the orientation *O_cj_*, we define the azimuth coefficient *ζ* as follows:

(1) $$\zeta =(2u-w)/w\left\{ \begin{array}{ll} \approx -1 & \text{left edge}\\ -1<\zeta <0 & \text{left side}\\ \approx 0 & \text{center}\\ 0<\zeta <1 & \text{right side}\\ \approx 1 & \text{right edge} \end{array}\right.$$

where *u* is the pixel coordinate of the center of the label in the horizontal direction, *w* is the total pixels of the image in the horizontal direction. 

**Figure 5:**
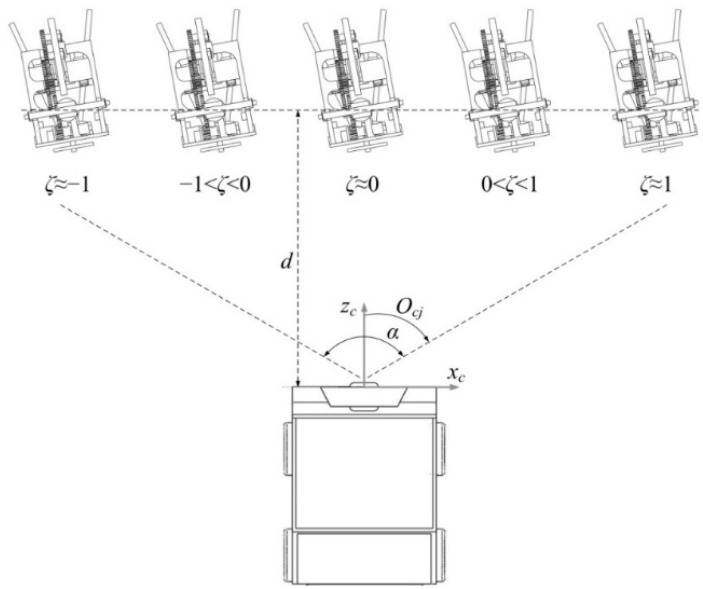
Diagram showing calculations of the azimuth coefficient *ζ* and orientation *O_cj_* of the carrier relative to the JSN.

The diagram of the azimuth coefficient *ζ* and orientation *O_cj_* calculations is shown in Figure 5. The orientation *O_cj_* and pitch angle *P_cj_* are as follows:

(2) $$\left\{ \begin{array}{l} O_{cj}=\arctan(x/d)\\ P_{cj}=\arctan(y/d) \end{array}\right.$$

where *d* is the distance between the JSN and the carrier, which is detected by the Kinect. *x* and *y* are the coordinates of the center of the label in the image plane coordinate frame as follows:

(3) $$\left\{ \begin{array}{l} x=2d\left(\frac{u}{R_{x}}-\frac{1}{2}\right)\tan(\frac{\alpha }{2})\\ y=-2d\left(\frac{v}{R_{y}}-\frac{1}{2}\right)\tan(\frac{\beta }{2}) \end{array}\right.$$

where *v* is the pixel coordinate of the center of the label in vertical direction; *R_x_* and *R_y_* are the resolutions of the images in *U* and *V* directions, respectively; and *α* and *β* are the angles of field of view of the Kinect in horizontal and vertical directions, respectively. *ζ* is used to decide the relative orientation roughly, while *O_cj_* is adopted to determine the exact relative orientation. Before *O_jc_* detecting, the carrier steers and calculates *ζ* and *O_cj_* until *ζ* ≈ 0 and *O_cj_* ≈ 0. 

### 3.2. Orientation of JSN Relative to Carrier

**Figure 6:**
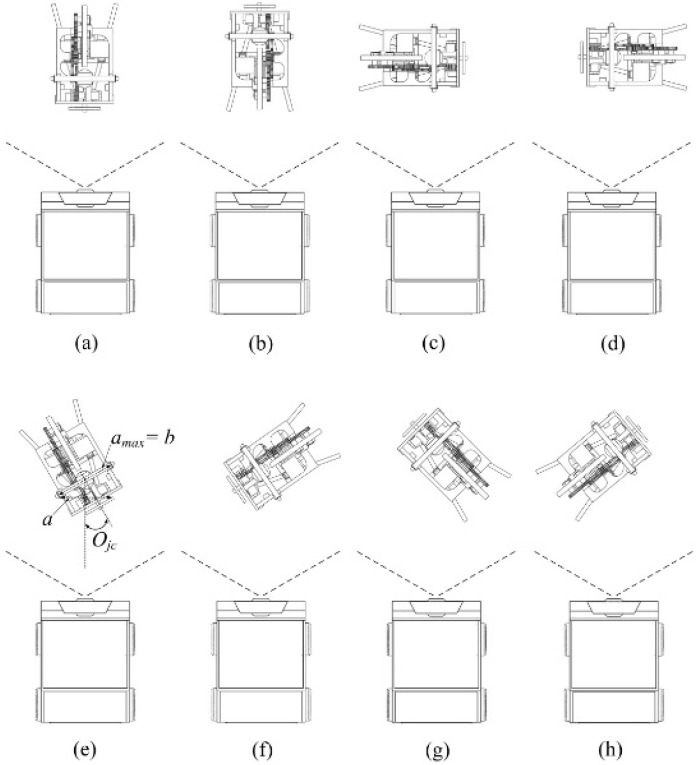
Eight conditions of the orientation of the JSN relative to the carrier: (**a**) JSN faces to the front side of the carrier; (**b**) JSN backs onto the front side of the carrier; (**c**) JSN faces to right side of the carrier; (**d**) JSN faces to left side of the carrier; (**e**) JSN faces to the front-right side of the carrier; (**f**) JSN faces to the front-left side of the carrier; (**g**) JSN backs onto the front-right side of the carrier; (**h**) JSN backs onto the front-left side of the carrier.

**Figure 7:**
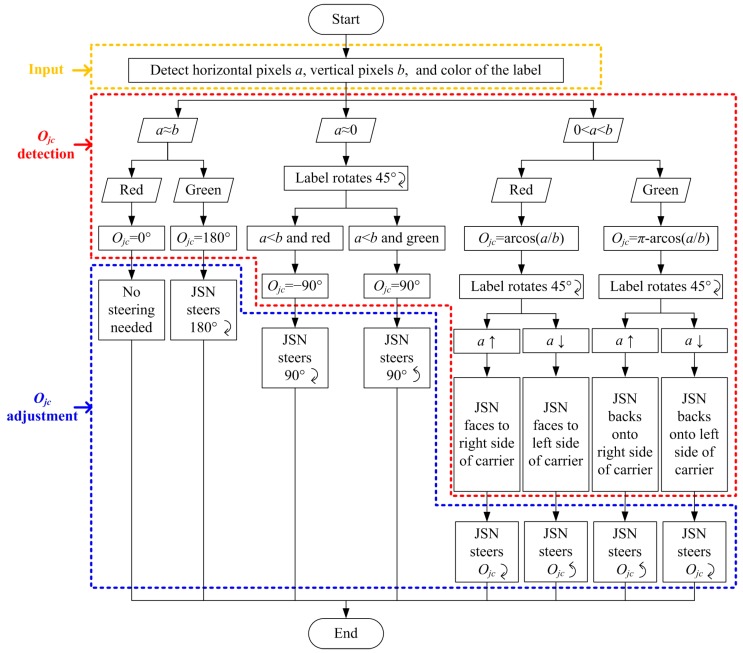
Algorithm for orientation *O_jc_* detection and adjustment of the JSN relative to the carrier.

After the carrier has detected and adjusted its orientation, the JSN is in the center of the images of the Kinect. In order to decide the orientation *O_jc_* of the JSN relative to the carrier, the two sides of the label are designed as red and green colors, respectively. In the beginning, the red side of the label faces to the front of the JSN. There are eight conditions for the *O_jc_* detection as illustrated in Figure 6. The algorithm for *O_jc_* detection and adjustment is shown in Figure 7. 

The first step for orientation (*O_jc_*) detecting is the color camera detects the horizontal pixels *a*, the vertical pixels *b*, and the color of the label which are the inputs of the algorithm. Then, the carrier judges the relationship between *a* and *b*, and the color to decide the conditions shown in Figure 6. If *a* ≈ *b* and the color is red, which is the condition shown in Figure 6a, the *O_jc_* is 0° and the JSN does not need to adjust its orientation. If *a* ≈ *b* and the color is green, which is the condition illustrated in Figure 6b, the *O_jc_* is 180° and the JSN needs to adjust its orientation 180° clockwise. If *a* ≈ 0, then the JSN controls its label to rotate 45° clockwise and the carrier judges the relationship between *a* and *b*, and the color of the label again. If *a* < *b* and the color is red, which is the condition shown in Figure 6c, the *O_jc_* is −90°, and the JSN needs to steer 90° clockwise. If *a* < *b* and the color is green, which is the condition shown in Figure 6d, the *O_jc_* is 90°, and the JSN has to steer 90° anticlockwise. 

If 0 < a < b, and the color is red, the orientation is:

(4) $$O_{jc}=\arccos(a/b)$$

then the label rotates 45° clockwise and the carrier judges the change trend of *a* in the initial stages. If *a* increases, it means that the JSN faces to the front-right side of the carrier, which is the condition shown in Figure 6e, and the JSN has to steer angle *O_jc_* clockwise. While if *a* decreases, it means that the JSN faces to the front-left side of the carrier, which is the condition shown in Figure 6f, and the JSN needs to steer angle *O_jc_* anticlockwise. If 0 < a < b, and the color is green, the orientation is:

(5) $$O_{jc}=\pi -\arccos(a/b)$$

Then the label rotates 45° clockwise and the carrier judges the change trend of *a* in the initial stages. If *a* increases, it implies that the JSN backs onto the front-right side of the carrier, which is the condition shown in Figure 6g, and the JSN has to steer angle *O_jc_* anticlockwise. While if *a* decreases, it indicates that the JSN backs onto the front-left side of the carrier, which is the condition shown in Figure 6h, and the JSN should steer angle *O_jc_* clockwise. After the JSN steers angle *O_jc_*, the front side of the JSN faces to the front side of the carrier. 

### 3.3. Position of JSN Relative to Carrier

**Figure 8:**
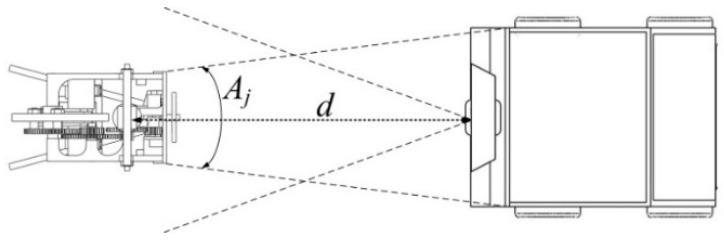
Diagram showing the relative distance detection.

The position of the JSN relative to the carrier can be decided when the carrier and the JSN face each other, as illustrated in Figure 8. The depth sensor of the Kinect detects the distance *d* between it and the label. Then the position of the JSN relative to the carrier is obtained. The carrier can move forward or backward to adjust the distance *d* in order to make the cabin be in the jumping range of the JSN. The JSN will jump into the cabin to realize the recycling. 

### 3.4. Dynamic Cooperation Strategies

Because the orientations and position detections may be influenced by some environment conditions and limited by the capability of the Kinect sensor, the dynamic cooperation between the JSN and the carrier is studied to improve the detection precisions. The dynamic cooperation includes three strategies. 

The first is that we assume the visual based orientations detection and the depth sensor based distance detection do not have very high precisions when the distance between the JSN and the carrier is too large. To deal with this problem, we can obtain the highest detection precisions at proper distances through experimental studies. The carrier is able to sense the JSN at a far distance such as 2 m with a low precision and then dynamically adjust their distance to enable the headings and distance detections have higher precisions, which are helpful for JSN recycling. 

The second one is that we assume there are objects having the similar colors and shapes with the label of the JSN in its surrounding. The carrier cannot distinguish the JSN from the objects correctly. The idea of dynamic cooperation to overcome this difficulty is that the label rotates to change its color and shape in the video images captured by the color camera. The differences between the images recorded before and after the rotation of the label can provide the clue for the carrier to find the JSN correctly. The size of the JSN is far smaller than the distances between the deployed JSNs. So, there is a small possibility that multiple JSNs are present in the field of view of the camera in the practical applications. In fact, we can also use the dynamic cooperation strategy to distinguish the JSN needed to be recycled from other JSNs if multiple JSNs are present in the field of view of the camera. The methodology is described as follows. The MAC addresses and node numbers of the JSNs are saved in the database of the carrier. The carrier broadcasts stop motion command to all the JSNs firstly. Then, the carrier sends label rotation command to the JSN needed to be recycled through its MAC address. After receiving the command and rotating its label, the JSN sends a reply message to the carrier. The carrier identifies the JSN from other JSNs by using the same method as from the objects resembling the colored label in the background of the JSN. If the labels of several JSNs overlap each other in the images, which almost the hardest condition for the carrier to identify, the carrier sends “dispersion” command to the JSNs to control them to jump one step. This could deal with the overlap problem. 

**Figure 9:**
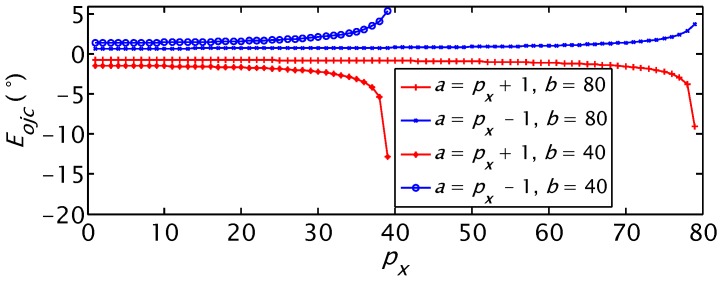
The relationship between *O_jc_* detection error and real horizontal pixels *p_x_*.

The third one is that the detection of *O_jc_* has different precisions at different real orientations of the label. From Equation (4) we can obtain the detection error *E_ojc_* of *O_jc_* in the range of (0°, 90°) when there is one pixel detection error of the label in the images:

(6) $$E_{ojc}=\arccos(a/b)-\arccos(p_{x}/b)$$

where 1 ≤ *p_x_* < (*b* − 1) is the real horizontal pixel of the label in the images, *a* = *p_x_* + 1 or *a* = *p_x_* − 1. Because *a* and *p_x_* decrease with the increase of *O_jc_*, as illustrated in Figure 6e, and the absolute value of *E_ojc_* increases with the increase of *p_x_* as shown in Figure 9, the absolute value of *E_ojc_* decreases with the increase of *O_jc_*. This means that *O_jc_* detection has higher precision when it is closer to 90°. The dynamic cooperation strategy is that the label rotates to a proper angle that is easier for the Kinect to detect the *O_jc_*. This will improve the precision of the orientation detection. The rotational angle of the label is recorded. The label rotates to its initial orientation after detecting *O_jc_*. 

## 4. Prototype Design and Fabrication

**Figure 10:**
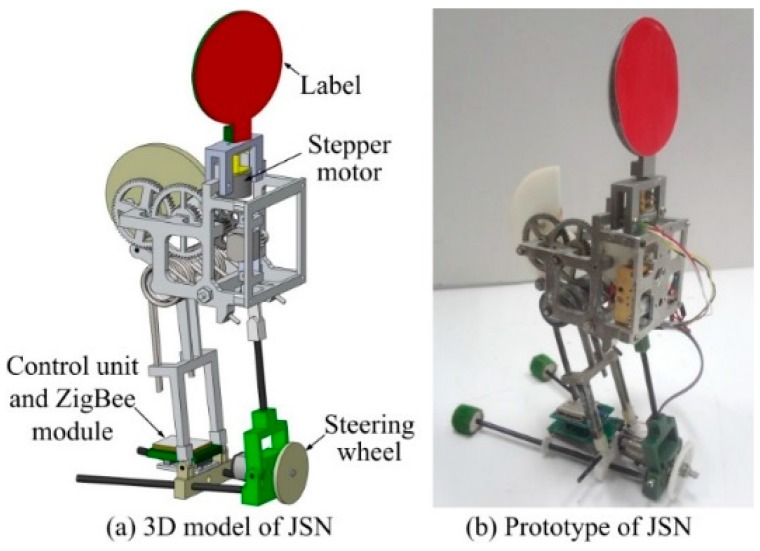
3D model and prototype of the JSN.

The 3D model and prototype of the JSN are shown in Figure 10. The size of the new prototype is about 10 cm × 7 cm × 17 cm. The JSN in this paper has the new steering mechanism and the disc-like label mechanism compared to our previous jumping robot [43,44]. The steering mechanism includes a steering wheel and a DC motor. The JSN can steer continuously when the wheel rotates driven by the motor. The step of the stepper motor is 2.4°. The control unit and the ZigBee wireless communication module can control the motions of the JSN, send data to the carrier and receive commands from it. The power of the JSN is supplied by a 3.7 V 200 mAh lithium battery. The total energy use of one jump of the JSN is about 1.53 mAh [44]. The energy use of the stepper motor in 360° rotation is about 0.053 mAh. So, the JSN could dock with the carrier using a little energy when its power is lower than the threshold. 

**Figure 11:**
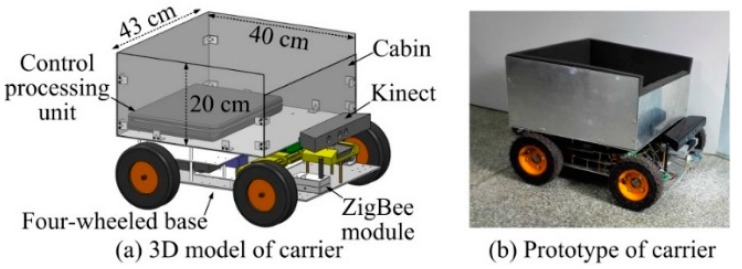
3D model and prototype of the carrier.

The 3D model and prototype of the carrier are shown in Figure 11. The four-wheeled base is driven by four servomotors. The Kinect mounted at the front side of the base has a color camera with selectable resolutions of 640 × 480 and 1280 × 960 and a depth camera with distance detection range about 0.6 m to 3.6 m. The field of view of the Kinect is 57° in horizontal direction and 43° in vertical direction. The 43 cm × 40 cm × 20 cm sized cabin for JSN recycling is installed on the top of the base. The angle *A_j_* is about 39.6° if the distance *d* is 0.5 m as shown in Figure 8. The control processing unit is mounted at the bottom of the cabin. The control unit is composed of a laptop and a four-axis control board. The laptop connects with the Kinect and runs the orientations and distance detection algorithm. The laptop also connects with the control board to control motions of the carrier. A ZigBee module on the front of the base is the coordinator forming the network with the JSN. 

## 5. Experimental Validations

The fundamental performances and influence factors on the detection precisions of the proposed relative orientation and position detection methods were tested firstly. The influence factors are shown in Table 1. The dynamic cooperation strategies were evaluated secondly. The JSN automatic detection of the prototype system was tested finally. 

**Table 1:** The influence factors in the experimental tests.

Symbol	Description
*d*	Distance between the carrier and JSN
*D*	Diameter of the label
*C*	Surface color to the label
*R*	Resolution of the color camera
*A*	Ambient illumination

### 5.1. Orientation Detection of Carrier Relative to JSN

The orientation detection of the carrier was tested at different distances *d*, different real orientations of *O_cj_*, and different orientations of *O_jc_* with a diameter *D* = 5 cm and surface color *C* = red label when ambient illumination *A* = 42 lux, resolution of the color camera *R* = 640 × 480, and the background was a white board. 

#### 5.1.1. *ζ* and *O_cj_* Detections at Different Distances *d*

**Figure 12:**
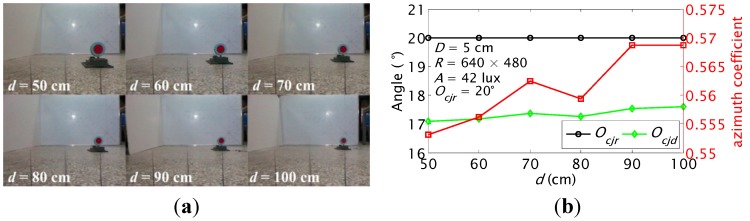
Azimuth coefficient and orientation detection results of the carrier at different distances when the real orientation *O_cjr_* is 20°: (**a**) recorded images; (**b**) calculated *ζ* and *O_cj_*.

*d* increased from 0.5 m to 1.0 m at the step of 0.1 m. The recorded images and calculated *ζ* and *O_cj_* are shown in Figure 12. The *ζ* increases slightly from 0.553 to 0.569. The *ζ* is larger than 0 and smaller than 1, which indicates that the label is in the right side of the images. The detected orientation *O_cjd_* increases from 17.1° to 17.6°. The results show that the highest detection precision can be obtained at distance of 1.0 m and the detection error is within 2.9°. 

#### 5.1.2. *ζ* and *O_cj_* Detections at Different *O_cjr_*

**Figure 13:**
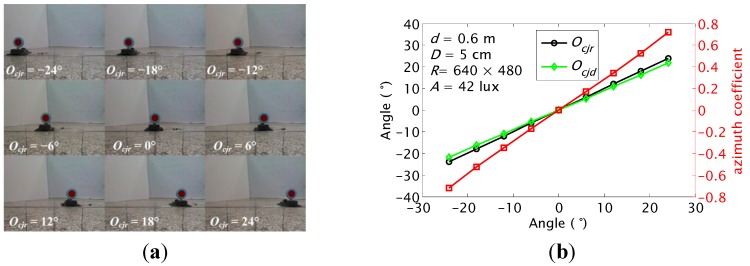
Azimuth coefficient and orientations detection results of the carrier at different real orientations when the distance *d* is 0.6 m: (**a**) recorded images; (**b**) calculated *ζ* and *O_cj_*.

The *ζ* and *O_cj_* were detected when the real orientation *O_cjr_* increased from ‒24° to 24° at the step of 6°. The results are shown in Figure 13. The *ζ* increases linearly with *O_cj_*. The *ζ* is utilized to decide the relative orientation roughly at different *O_cjr_*. The largest error of *O_cj_* detection is only about 2.22° when |*O_cjr_*| = 24°. The results show that the proposed method can detect the orientation *O_cj_* with a high precision for JSNs recycling. 

#### 5.1.3. *ζ* and *O_cj_* Detections at Different *O_jc_*

**Figure 14:**
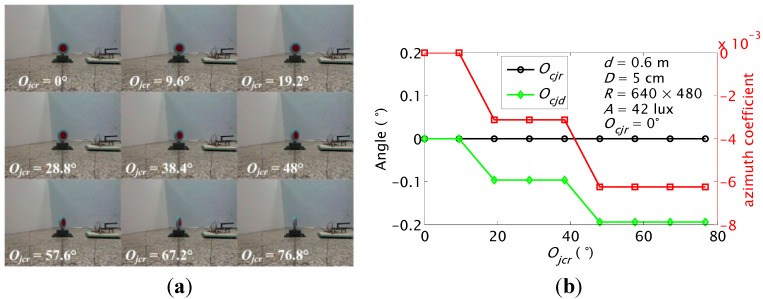
Azimuth coefficient and orientation detection results of the carrier when the orientation of the JSN is set as different values: (**a**) recorded images; (**b**) calculated *ζ* and *O_cj_*.

In this test, orientation *O_jcr_* of the JSN relative to the carrier increased from 0° to 76.8° at the step of 9.6° when the real orientation *O_cjr_* was set as 0°. The results are shown in Figure 14. The *O_cj_* detection has highest precision when *O_jcr_* is 0° and 9.6°. The precision decreases with the increase of the *O_jcr_*. The largest error is ‒0.19°. The carrier cannot detect the JSN when the *O_jcr_* is close to 90°. 

### 5.2. Orientation Detection of JSN Relative to Carrier

**Figure 15:**
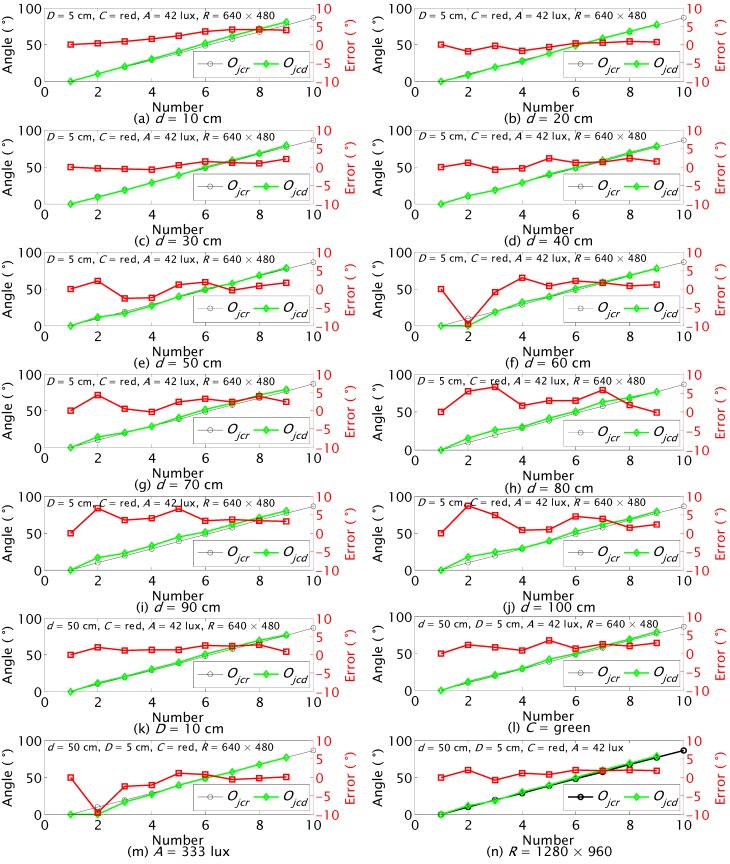
*O_jc_* detection results at different distances *d*, diameters of the label *D*, and colors of the label *C*, ambient illuminations *A* and resolutions of the color camera *R*.

Because the orientation detection of the JSN was based on the horizontal and vertical pixels sensing of the label in the images, the influence factors shown in Table 1 were tested in this section. The detection results at different *d*, *D*, *C*, *A* and *R* are shown in Figure 15. The label rotated from 0° to 86.4° at the step of 9.6°. 

The real orientation *O_jcr_* and the detected orientation *O_jcd_* at different *d* are shown in Figure 15a–j. The largest errors at different *d* mainly happen when the real orientation is near 9.6°. The results show that the proposed method can detect the orientation of the label with an acceptable precision at different distances. The detection precision is higher at smaller distance and when the real orientation *O_jcr_* is close to 90°. 

The largest errors are −2.6° and 2.69° when the diameters *D* of the labels are 5 cm and 10 cm as shown in Figure 15e,k, respectively. The results indicate that the detection error of larger label is not smaller than the error of the smaller label. The 5 cm label will be installed into the JSN for recycling test because the size of the JSN should be as small as possible. 

The red and green labels were tested. The largest errors for the orientation detections of the red and green labels are −2.6° and 3.5° as shown in Figure 15e,i, respectively. The results indicate that both the red and green labels are easier to be detected for the JSN sensing. 

The ambient illumination was changed with different number of lights in the indoor environment. The tests were performed at *A* = 42 lux and *A* = 333 lux as detected by a light sensor TSL2550. The results are shown in Figure 15e,m. The largest errors are ‒2.6° and −9.6°, respectively. The results indicate that too strong light of the environment has influences on the detection precision. 

The resolution of the camera was set as 640 × 480 and 1280 × 960 for influence factor *R* tests. The results are shown in Figure 15e,n. The largest errors are −2.6° and 2.0°, respectively. The results indicate that higher detection precision can be obtained at higher resolution of the camera. 

### 5.3. Position Detection of JSN Relative to Carrier

The position of the JSN relative to the carrier could be decided after the orientations and distance between the JSN and carrier are detected. The distance detection tests were conducted at different real distances, sizes and colors of the label, and ambient illuminations when the Kinect faced to the JSN. 

The test results at different real distances *D_r_* are shown in Table 2. The detected distances *D_d_* are zeros when the *D_r_* are 40 cm and 50 cm. This indicates that the depth sensor of the Kinect cannot detect too small distances. The largest error is −3.1 cm when the *D_r_* is 200 cm. The test results at different *D*, *C*, and *A* when the real distance *D_r_* = 70 cm are shown in Table 3. 

The results of distance detection show that the maximum error is only about 3 cm within 200 cm detection range, and the detection precision is higher when the real distance is smaller. The size and color of the label and the ambient illumination do not have serious influences on the distance detection precision. The errors are within 1 cm. 

**Table 2:** Distance between the JSN and the carrier detection results when the real distance increases from 40 cm to 200 cm.

Name	Real and Detected Distances (cm)									Conditions
*D_r_* (cm)	40	50	60	70	80	90	100	110	120	*D* = 5 cm, *C* = red, *A* = 42 lux
	130	140	150	160	170	180	190	200		
*D_d_* (cm)	0	0	60.3	70.1	80.2	90.1	100	109.5	119.3	
	128.7	139	149.2	158.9	169	178.6	188.3	196.9		

**Table 3:** Distance detection results at different diameters of the label *D*, colors of the label *C*, and ambient illuminations *A* when the real distance *D_r_* = 70 cm.

Detected Distance *D_d_* (cm)	Conditions
70.1	*D* = 5 cm, *C* = red, *A* = 42 lux
70.5	*D* = 10 cm, *C* = red, *A* = 42 lux
70.1	*D* = 5 cm, *C* = green, *A* = 42 lux
70.3	*D* = 5 cm, *C* = red, *A* = 121 lux
70.3	*D* = 5 cm, *C* = red, *A* = 286 lux

### 5.4. Dynamic Cooperation between JSN and Carrier

#### 5.4.1. Dynamic Cooperation for Detection of Distance *d*

**Figure 16:**
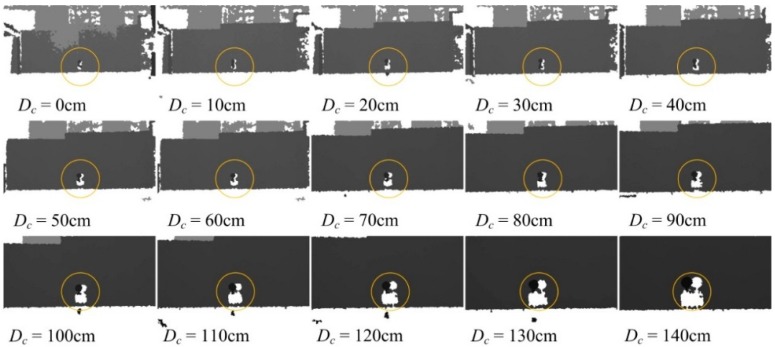
Depth images at different controlled displacements in the dynamic cooperative distance detection.

The initial distance between the JSN and the carrier was set as 200 cm. The carrier moved to the JSN at a step of 10 cm and detected the label periodically. The carrier recorded the distance between it and the JSN at every step. The real displacement of the carrier was measured. The depth images at different controlled displacements *D_c_* are shown in Figure 16. The black background is the board behind the JSN. The recognized JSN is circled by orange circles. The carrier stopped moving to the JSN at *D_c_* of 140 cm. 

**Figure 17:**
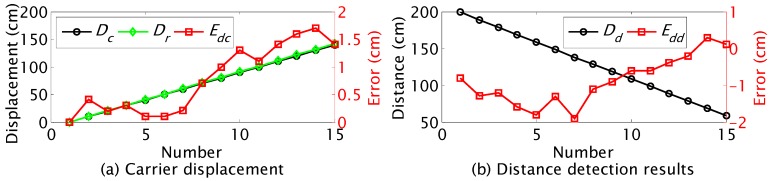
Results of dynamic cooperation for distance detection.

The controlled displacement *D_c_*, the real displacement *D_r_*, and the displacement control error *E_dc_* are shown in Figure 17a. The error *E_dc_* has the trend of increase with the displacement of the carrier. The maximum error is about 1.7 cm. The detected distance *D_d_* and the detection error *E_dd_* are shown in Figure 17b. The absolute value of *E_dd_* decreases with displacement of the carrier. The absolute value of *E_dd_* is reduced from 1.9 cm to about 0.1 cm. This indicates that the distance detection precision could be improved by using the dynamic cooperation strategy. 

#### 5.4.2. Dynamic Cooperation for JSN Detecting

**Figure 18:**
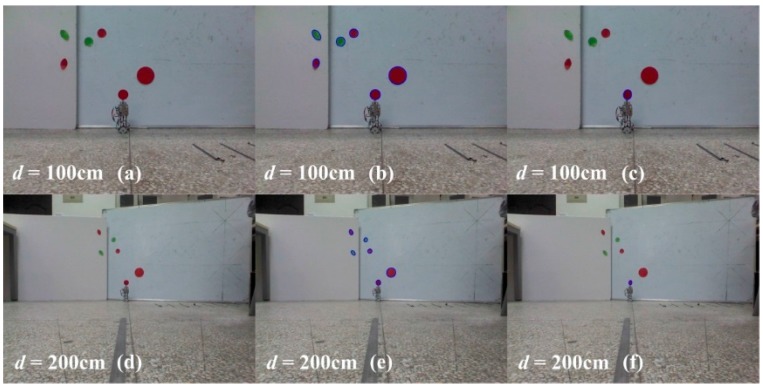
Test results of the carrier detecting the JSN when there are similar shaped and same colored interferences in the surrounding of the label. (**a**,**d**) are the images before detection; (**b**,**e**) are the images when all the red and green circles and ellipses are detected; (**c**,**f**) are the images after the label rotates a proper angle and the JSN is distinguished from the interferences.

Several circle and ellipse like labels with the similar size and same color of the label of the JSN were stuck on the white board to imitate the situation that there were objects or other JSNs in the background of the JSN needed to be recycled. The board was put behind the JSN. The carrier steered to detect the labels. The carrier stopped steering and recorded all the sizes, shapes, and colors of the labels when it found the labels. Then it sent a control command to the JSN. The label on the JSN rotated a proper angle to adjust its shape on the images of the Kinect. The carrier detected the labels again and calculated the differences between the images recorded before and after the rotation of the label to distinguish the label of JSN from other labels. The recorded and processed images are shown in Figure 18. The results show that the carrier is able to distinguish the JSN from the interferences successfully when *d* is 100 cm and 200 cm. This capability improves the JSN detection success rate for recycling. 

#### 5.4.3. Dynamic Cooperation for Detection of Orientation *O_jc_*

**Figure 19:**
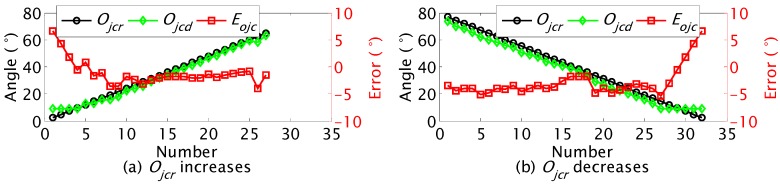
Results of dynamic cooperation for detection of *O_jc_*.

The real orientation of the JSN was controlled to increase from 2.4° to 64.8° at the step of 2.4°. The carrier detected the orientation of the JSN at every step and calculated the detection error. The real orientation *O_jcr_*, the detected orientation *O_jcd_*, and the orientation detection error *E_ojc_* are shown in Figure 19a. The error decreases from 6.6° to 0.83°. The variation trend agrees with the simulation results shown in Figure 9. This verifies that we are able to obtain higher detection precision using the dynamic cooperation strategy. The real orientation was also controlled to decrease from 78.8° to 2.4° at the step of 2.4°. The error shown in Figure 19b also gives validation of the proposed dynamic cooperation strategy. 

### 5.5. JSN Automatic Detection Test

**Figure 20:**
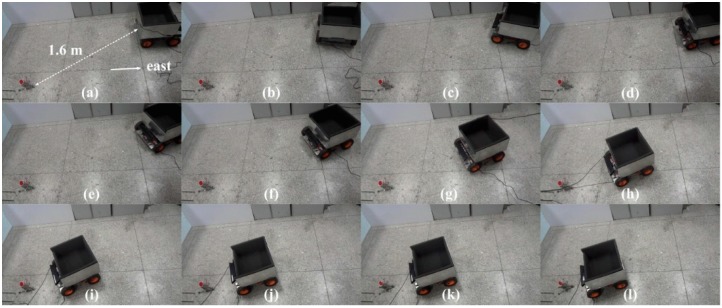
Video sequences of the orientations and position automatic detection process: (**a**) initial condition; (**b**) carrier steers anticlockwise about 80°; (**c**) carrier steers continuously; (**d**) carrier finds the JSN is in the video images; (**e**) carrier steers and calculates *ζ* and *O_cj_* step by step until they are close to zero; (**f**) carrier moves to the JSN; (**g**) carrier moves to the JSN continuously; (**h**) carrier moves to the JSN continuously; (**i**) carrier stops in front of the JSN; (**j**) carrier detects orientation *O_jc_*; (**k**) JSN steers angle *O_jc_*; (**l**) carrier moves to the JSN again.

The performance of the prototype system for JSN automatic detection was tested finally. In the beginning of the test, the JSN was put behind the carrier on the right 1.6 m far, and the headings of the carrier and JSN were east. The carrier ran the detection algorithm and dynamic cooperated with the JSN. The video sequences of the orientations and position detection process are shown in Figure 20. The carrier stared to steer anticlockwise and tried to find if the JSN is in the video images. After finding the JSN in the images, the carrier calculated and recorded the azimuth coefficient *ζ* and the orientation *O_cj_* step by step. When the *ζ* and *O_cj_* were close to zero, the carrier stopped to steer and began to move to the JSN. The carrier stopped in front of the JSN at the distance about 30 cm and began to detect the orientation *O_jc_* of the JSN. The detected *O_jc_* was about 20.2°. The carrier sent the *O_jc_* to the JSN. The JSN steered angle *O_jc_* when it faced to the carrier. The carrier moved to the JSN again and stopped when the distance *d* was about 20 cm. Then the JSN could jump into the cabin for recycling. 

## 6. Conclusions and Future Work

We propose short-distance relative orientation and position detection methods between a carrier robot and jumping sensor nodes during JSNs recycling. The methods are based on the RGB-D sensor and the dynamic cooperation strategies. The system components, the recycling procedure, and the detection methods are introduced, respectively. A prototype system including a carrier and a JSN are designed and fabricated for validating the proposed methods. The orientations and position of the carrier and the JSN relative each other are tested at different situations. The results show that the orientation detection of the carrier relative to the JSN has largest errors about 3° at different test distances, different real headings, and different orientation of the JSN. The orientation detection tests of the JSN show that higher precision can be obtained at smaller distance. The size and color of the label do not have serious influences on the detection precision. But the precision decreases when the ambient illumination increases. This is because the preset thresholds of the RGB values are static during the tests while the ambient illumination has effects on the RGB values of the images. The higher detection precision of this orientation could be obtained when the Kinect is set at higher resolution. The distance detection results show that the maximum error is only about 3 cm within 200 cm detection range, and the detection precision is higher when the real distance is smaller. The dynamic cooperation strategies test results show that the distance detection error could be reduced from several centimeters to several millimeters. This error is far smaller than the jumping range of the JSN. The carrier is able to distinguish the JSN from the interferences successfully when the distance between them is as far as 2 m, which improves the success rate of the JSN detection for recycling. The results also show that the orientation detection of the JSN has higher precision when the real orientation is close to 90°. The detection error could be reduced from 6.6° to about 1°, which is far smaller than angle *A_j_* (39.6°), so the JSN could jump into the cabin easily. The JSN automatic detection test results show that the carrier can detect and move to the JSN successfully and the JSN could be recycled after it jumps into the cabin. 

The proposed detection methods combined with the dynamic cooperation strategies have high detection precisions for JSNs recycling. The proposed methods in this paper could not only be used for JSNs, but also be adopted for other kinds of mobile sensor nodes and multi-robot systems. The limitations include that the visual based detection methods could be affected by the ambient illumination and the performance of the infrared-based depth sensor of the Kinect could significantly be affected by the sunlight in outdoor application environment. 

Future work includes four main aspects. Firstly, the factors that influence visual based orientation detection method will be dynamically compensated using light sensor to improve the detection precision. Furthermore, the ultrasonic sensor or laser sensor could be combined with infrared distance sensor for application in outdoor environment with strong sunlight. Secondly, the long-distance localization methods and multisensor data fusion will be investigated for the JSNs recycling. Thirdly, we will try to design docking method for the suddenly damaged JSNs, which cannot jump into the cabin of the carrier. A simple and low cost manipulator combined with the visual detection method could be a solution for this problem. Finally, the universality of the proposed methods for JSNs recycling will be tested on other kinds of miniature robotic mobile sensor nodes. 
